# The Bitcoin as a Virtual Commodity: Empirical Evidence and Implications

**DOI:** 10.3389/frai.2020.00021

**Published:** 2020-04-30

**Authors:** Cinzia Baldan, Francesco Zen

**Affiliations:** Department of Economics and Management, University of Padova, Padua, Italy

**Keywords:** Bitcoin, FinTech, Vector Autoregression model, distributed ledger technology, cryptocurrencies price determination, G12, C52, D40

## Abstract

The present work investigates the impact on financial intermediation of distributed ledger technology (DLT), which is usually associated with the blockchain technology and is at the base of the cryptocurrencies' development. “Bitcoin” is the expression of its main application since it was the first new currency that gained popularity some years after its release date and it is still the major cryptocurrency in the market. For this reason, the present analysis is focused on studying its price determination, which seems to be still almost unpredictable. We carry out an empirical analysis based on a cost of production model, trying to detect whether the Bitcoin price could be justified by and connected to the profits and costs associated with the mining effort. We construct a sample model, composed of the hardware devices employed in the mining process. After collecting the technical information required and computing a cost and a profit function for each period, an implied price for the Bitcoin value is derived. The interconnection between this price and the historical one is analyzed, adopting a Vector Autoregression (VAR) model. Our main results put on evidence that there aren't ultimate drivers for Bitcoin price; probably many factors should be expressed and studied at the same time, taking into account their variability and different relevance over time. It seems that the historical price fluctuated around the model (or implied) price until 2017, when the Bitcoin price significantly increased. During the last months of 2018, the prices seem to converge again, following a common path. In detail, we focus on the time window in which Bitcoin experienced its higher price volatility; the results suggest that it is disconnected from the one predicted by the model. These findings may depend on the particular features of the new cryptocurrencies, which have not been completely understood yet. In our opinion, there is not enough knowledge on cryptocurrencies to assert that Bitcoin price is (or is not) based on the profit and cost derived by the mining process, but these intrinsic characteristics must be considered, including other possible Bitcoin price drivers.

## Introduction

A strict definition of FinTech seems to be missing since it embraces different companies and technologies, but a wider one could assert that FinTech includes those companies that are developing new business models, applications, products, or process based on digital technologies applied in finance.

Financial Stability Board (FSB) (2017) defines FinTech as “technology-enabled innovation in financial services that could result in new business models, applications, processes, or products with an associated material effect on the provision of financial services.”

OECD (2018) analyzes instead various definitions from different sources, concluding that none of them is complete since “FinTech involves not only the application of new digital technologies to financial services but also the development of business models and products which rely on these technologies and more generally on digital platform and processes.”

The services offered by these companies are indeed various: some are providing financial intermediation services (FinTech companies), while others offer ancillary services relating to the financial intermediation activity (TechFin companies). Technology is, for FinTech firms, an instrument, a productive factor, an input, while for TechFin firms, it is the final product, the output. The latter are already familiar with different technologies and innovation; hence, they could easily diversify their production by adding some digital and financial services to the products they already offer. They enjoy a situation of privileged competition because they are already known in the market due to their previous non-financial services and thus could take advantage of their customers' information to enlarge their supply of financial services. TechFin firms are the main competitors for FinTech companies (Schena et al., 2018). Indeed FinTech, or financial technology, is changing the way in which financial operations are carried out by introducing new ways to save, borrow, and invest, without dealing with traditional banks.

FinTech platforms, firms, and startups rose after the global financial crisis in 2008 as a consequence of the loss of trust in the traditional financial sector. In addition, digital natives (or millennials, born between 1980 and 2000) seemed interested in this new approach proposed by FinTech entrepreneurs. Millennials were old enough to be potential customers, who feel much more related to these new, fresh mobile services offered through mobile platforms and apps, rather than bankers. The strength of these new technologies lies in their transparent and easy-to-use interfaces that was seen as an answer to the trust crisis toward banks (Chishti and Barberis, 2016).

After the first Bitcoin (Nakamoto, 2008) has been sent in January 2009, hundreds of new cryptocurrencies started being traded in the market, whose common element is to rely on a public ledger (or blockchain technology; Hileman and Rauchs, 2017). In fact, in addition to Bitcoin, other cryptocurrencies gained popularity, such as: Ethereum (ETH), Dash, Monero (XMR), Ripple (XRP), and Litecoin (LTC). Ethereum (ETH) was officially launched in 2015 and is a decentralized computing platform characterized by its own programming language. Dash was introduced in 2014 but its market value was rising in 2016. The peculiarity of this digital coin is that, in contrast with other cryptocurrencies, block rewards are equally shared among community participants and a revenue percentage (equal to 10%) is stored in the “treasury” to fund further improvements, marketing, and network operations. Monero (XMR), launched in 2014, is a system that guarantees anonymous digital cash by hiding the features of the transacted coins. Its market value raised in 2016. Ripple (XRP) has the unique feature to be based on a “global consensus ledger” rather than on blockchain technology. Its protocol is adopted by large institutions like banks and money service businesses. Litecoin (LTC) appeared for the first time in 2011 and is characterized by a large supply of 84 million LTC. Its functioning is based on that of Bitcoin, but some parameters were altered (the mining algorithm is based on Scrypt rather than Bitcoin's SHA-265).

Despite the creation of these new cryptocurrencies, Bitcoin remains the main coin in terms of turnover. The main advantage of this new digital currency seems to be the low cost of transaction (even if this is actually a myth, since BTC transactions topped out at 50 USD per transaction in 2017–2018, while private banks charge less these days) and, contrary on what many people think, anonymity was not one of its main features when this network was designed. An individual could attempt to make his identity less obvious but the evidences available by now do not support the claim that it could be hidden easily; it may be probably impossible. To this purpose, fiat physical currencies remain the best option.

Hayes (2015, 2017, 2019) analyzes the Bitcoin price formation. In particular, he assumes the cryptocurrency as a virtual commodity, starting from the different ways by which an individual could obtain it. A person could buy Bitcoins directly in an online marketplace by giving in exchange fiat currencies or other types of cryptocurrencies. Alternatively, he can accept them as payment and finally an individual can decide to “mine” Bitcoins, which consists in producing new units, by using computer hardware designed for this purpose. This latter case involves an electrical consumption and a rational agent would not be involved in the mining process if the marginal costs of this operation exceed its marginal profits. The relation between these values determines price based on the cost of production that is the theoretical value underlying the market price, around which it is supposed to gravitate. Abbatemarco et al. (2018) resume Hayes' studies introducing further elements missed in the previous formulation. The final result confirms Hayes' findings: the marginal cost model provides a good proxy for Bitcoin market price, but the development of a speculative bubble is not ruled out.

We study the evolution of Bitcoin price by considering a cost of production model introduced by Hayes (2015, 2017, 2019). Adding to his analysis some adjustment proposed by Abbatemarco et al. (2018), we recover a series for the hypothetical underlying price; then, we study the relationship between this price and the historical one using a Vector Autoregression (VAR) model.

The remainder of the paper proceeds as follows: in section Literature Review, we expose a literature overview, presenting those papers that investigate other drivers for Bitcoin price formation, developing alternative approaches. In section Materials and Methods, we exploit the research question, describing the methodology behind the implemented cost of production model, the sources accessed to collect data, the hardware sample composition, and the formula derivations. In section Main Outcomes, we analyze and comment on the main findings of the analysis; section Conclusions concludes the work with our comments on main findings and their implications.

## Literature Review

Researchers detect a number of economic determinants for Bitcoin price; it seems that given the new and particular features of this cryptocurrency, price drivers will change over time. For this reason, several authors analyze various potential factors, which encompass technical aspects (such as the hashrate and output volume), user-based growth, Internet components (as Google Trends, Wikipedia queries, and Tweets), market supply and demand, financial indexes (like S&P500, Dow Jones, FTSE100, Nikkei225), gold and oil prices, monetary velocity, and exchange rate of Bitcoin expressed in US dollar, euro, and yen. Among others, Kristoufek (2015) focuses on different sources of price movements by examining their interconnection during time. He considers different categories: economic drivers, as potential fundamental influences, followed by transaction and technical drivers, as influences on the interest in the Bitcoin. The results show how Bitcoin's fundamental factors, such as usage, money supply and price level, drive its price over the long term. With regard to the technical drivers, a rising price encourages individuals to become miners but this effect eclipses over time, since always more specialized mining hardware have increased the difficulty. Evidences show that price is even driven by investors' interest. According to previous studies (Kristoufek, 2013; Garcia et al., 2014), the relationship appears as most evident in the long run, but during episodes of explosive prices, this interest drives prices further up, while during rapid declines, it pushes them further down. He then concludes that Bitcoin is a unique asset with properties of both a speculative-financial asset, and a standard one and because of his dynamic nature and volatility, it is obvious to expect that its price drivers will change over time. The interest element seems to be particularly relevant when analyzing the behavior of Bitcoin price, leading many researchers to study its interconnection with Internet components, such as Google Trends, Wikipedia queries, and Tweets.

Even Matta et al. (2015) investigate whether information searches and social media activities could predict Bitcoin price comparing its historical price to Google Trends data and volume of tweets. They used a dataset based only on 60 days, but, in addition to the other papers regarding this topic, they implement an automated sentiment analysis technique that allows one to automatically identify users' opinions, evaluations, sentiments, and attitudes on a particular topic. They use a tool called “SentiStrength,” which is based on a dictionary only made by sentiment words, where each of them is linked to a weight representing a sentiment strength. Its aim is to evaluate the strength of sentiments in short messages that are analyzed separately, and the result is summed up in a single value: a positive, negative, or neutral sentiment. The study reveals a significant relationship between Bitcoin price and volumes of both tweets and Google queries.

Garcia et al. (2014) study the evolution of Bitcoin price based on the interplay between different elements: historical price, volume of word-of-mouth communication in on-line social media (information sharing, measured by tweets, and posts on Facebook), volume of information search (Google searches and Wikipedia queries), and user base growth. The results identify an interdependence between Bitcoin price and two signals that could form potential price bubbles: the first concerns the word-of-mouth effect, while the other is based on the number of adopters. The first feedback loop is a reinforcement cycle: Bitcoin interest increases, leading to a higher search volume and social media activity. This new popularity encourages users to purchase the cryptocurrency driving the price further up. Again, this effect would raise the search volume. The second loop is the user adoption cycle: after acquiring information, new users join the network, growing the user base. Demand rises but since supply cannot adjust immediately but changes linearly with time, Bitcoin price would increase.

Ciaian et al. (2016) adopt a different approach to identify the factors behind the Bitcoin price formation by studying both the digital and traditional ones. The authors point out the relevance of analyzing these factors simultaneously; otherwise, the econometric outputs could be biased. To do so, they specify three categories of determinants: market forces of supply and demand; attractiveness indicators (views on Wikipedia and number of new members and posts on a dedicated blog), and global macro-financial development. The results show that the relevant impact on price is driven by the first category and it tends to increase over time. About the second category, they assert that the short-run changes on price following the first period after Bitcoin introduction are imputable to investors' interest, which is measured by online information search. Its impact eases off during time, having no impact in the long run and may be due to an increased trust among users who become more willing to adopt the digital currency. On the other hand, the results suggest that investor speculations can also affect Bitcoin price, leading to a higher volatility that may cause price bubbles. To conclude, the study does not detect any correspondences between Bitcoin price and macroeconomics and financial factors.

Kjærland et al. (2018) try to identify the factors that have an impact on Bitcoin price formation. They argue that the hashrate, CBOE volatility index (VIX), oil, gold, and Bitcoin transaction volume do not affect Bitcoin price. The study shows that price depends on the returns on the S&P500, past price performance, optimism, and Google searches.

Bouoiyour and Selmi (2015) examine the links between Bitcoin price and its potential drivers by considering investors' attractiveness (measured by Google search queries); exchange–trade ratio; monetary velocity; estimated output volume; hashrate; gold price; and Shanghai market index. The latter value is due to the fact that the Shanghai market is seen as the biggest player in Bitcoin economy, which could also drive its volatility. The evaluation period is the one from 5th December 2010 to 14th July 2014 and it is investigated through the adoption of an ARDL Bounds Testing method and a VEC Grander causality test. The results highlight the speculative nature of this cryptocurrency stating that there are poor chances that it becomes internationally recognized.

Giudici and Abu-Hashish (2019) propose a model to explain the dynamics of bitcoin prices, based on a correlation network VAR process that models the interconnections between different crypto and classic asset price. In particular, they try to assess whether bitcoin prices in different exchange markets are correlated with each other, thus exhibiting “endogenous” price variations. They select eight exchange markets, representative of different geographic locations, which represent about 60% of the total daily volume trades. For each exchange market, they collect daily data for the time period May 18th, 2016 to April 30th, 2018. The authors also try to understand whether bitcoin price variations can also be explained by exogenous classical market prices. Hence, they use daily data (market closing price) on some of the most important asset prices: gold, oil, and SP500, as well as on the exchange rates USD/Yuan and USD/Eur. Their main empirical findings show that bitcoin prices from different exchanges are highly interrelated, as in an efficiently integrated market, with prices from larger and/or more connected trading exchanges driving the others. The results also seem to confirm that bitcoin prices are typically unrelated with classical market prices, thus bringing further support to the “diversification benefit” property of crypto assets.

Katsiampa (2017) uses an Autoregressive model for the conditional mean and a first-order GARCH-type model for the conditional variance in order to analyze the Bitcoin price volatility. The study collects daily closing prices for the Bitcoin Coindesk Index from 18th July 2010 to 1st October 2016 (2,267 observations); the returns are then calculated by taking the natural logarithm of the ratio of two consecutive prices. The main findings put on evidence that the optimal model in terms of goodness of fit to the data is the AR-CGARCH, a result that suggests the importance of having both a short-run and a long-run component of conditional variance.

Chevallier et al. (2019) investigate the Bitcoin price fluctuations by combining Markov-switching models with Lévy jump-diffusion to match the empirical characteristics of financial and commodity markets. In detail, they try to capture the different sub-periods of crises over the business cycle, which are captured by jumps, whereas the trend is simply modeled under a Gaussian process. They introduce a Markov chain with the existence of a Lévy jump in order to disentangle potentially normal economic regimes (e.g., with a Gaussian distribution) vs. agitated economic regimes (e.g., crises periods with stochastic jumps). By combining these two features, they offer a model that captures the various crashes and rallies over the business cycle, which are captured by jumps, whereas the trend is simply modeled under a Gaussian framework. The regime-switching Lévy model allows identifying the presence of discontinuities for each market regime, and this feature constitutes the objective of the proposed model.

## Materials and Methods

We study the evolution of Bitcoin price by considering a cost of production model introduced by Hayes (2015, 2017). Adding to his analysis some adjustment proposed by Abbatemarco et al. (2018), we recover a series for the hypothetical underlying price, and we study the relationship between this price and the historical one using a VAR model. In detail, Hayes back-tests the pricing model against the historical market price to consolidate the validity of his theory. The findings show how Bitcoin price is significantly described by the cryptocurrency's marginal cost of production and suggest that it does not depend on other exogenous factors. The conclusion is that during periods in which price bubbles happen, there will be a convergence between the market price and the model price to shrink the discrepancy. Abbatemarco et al. (2018) resume Hayes' studies introducing further elements missed in the previous formulation. The final result confirms Hayes' findings: the marginal cost model provides a good proxy for Bitcoin market price, but the development of a speculative bubble is not ruled out. Since these studies were published before Bitcoin price raise reached its peak on 19th December 2017 (the value was $19,270), the aim of our work is to extend the analysis considering a larger time frame and verify if, even in this case, the results are unchanged. In particular, we consider the period from 9th April 2014 to 31st December 2018. We start with some unit root tests to verify if the series are stationary in level or need to be integrated and then we identify the proper number of lags to be included in the model. We then check for the presence of a cointegrating relationship to verify whether we should adopt a Vector Error Correction Model (VECM) or a VAR model; the results suggest that a VAR model is the best suited for our data^1^. We thus collect the final results of the analysis and we improve them by correcting the heteroscedasticity in the regressions.

The marginal cost function, which estimates the electrical costs of the devices used in the mining process, is presented as Equation (1):

(1) $$\begin{array}{l} COST_{\frac{\$}{day}}=\;H_{\frac{hash}{s}}\;*\;Eff_{\frac{J}{hash}}\;*\;CE_{\frac{\$}{kWh}}\;*\;24_{\frac{h}{day}} \end{array}$$

Where:

*H*_*hash*/*s*_ is the hashrate (measured by hash/second);
*EFF*_*J*/*hash*_ is the energy efficiency of the devices involved in the process and it is measured by Joule/hash;
*CE*_*$*/*kWh*_ is the electricity cost expressed in US dollar per kilowatt/hour;
24 is the number of hours in a day;

A marginal profit function, which estimates the reward of the mining activity, is instead depicted as Equation (2):

(2) $$\begin{array}{l} PROFIT_{\frac{BTC}{day}}=BR_{BTC}\;*\;\left[\frac{{3,600}_{\frac{s}{h}}\;*\;24_{\frac{h}{day}}}{BTs}\right] \end{array}$$

Where:

*BR*_*BTC*_ is the block reward that refers to new Bitcoins distributed to miners who successfully solved a block (hence it is measured by BTC) and it is given by a geometric progression (Equation 3):

(3) $$\begin{array}{l} BR_{BTC}=BR_{1}\;*\;{\frac{1}{2}}^{n-1} \end{array}$$

*n* increases by 1 every 210,000 blocks. At the beginning, it was *BR*_1_ = 50, but during the course of time, it halved twice: on 29th November 2012 and on 10th July 2016.

3,600 is the number of seconds in an hour;

24 is again the number of hours in a day;

*BT*_*s*_ is the block time, which is expressed as the seconds needed to generate a block (around 600 s = 10 min), and it is computed as Equation (4):

(4) $$\begin{array}{l} BT_{s}=\;\frac{D\;*\;2^{32}}{H} \end{array}$$

Where *H* = hashrate and *D* = difficulty. The latter variable specifies how hard it is to generate a new block in terms of computational power given a specific hashrate. This is the value that changes frequently to ensure a *BT*_*s*_ close to 10 min^2^.

In addition to the variables already considered, we introduce some adjustments proposed by Abbatemarco et al. (2018), who thought there were two elements missing in Hayes' formulations.

They add, on the cost side, the one required to maintain and update miners' hardware (MAN, expressed in US dollar), and on the profit side, the fees (FEES) received by miners who place transactions in a block^3^.

Maintenance costs are computed as a ratio between the weighted devices' price and their weighted lifespan (5), while fees, expressed in BTC, are measured as a ratio between the daily total transaction fees and the number of daily transactions^4^ (6).

(5) $$\begin{array}{l} MAN_{\$}\;=\frac{Weighted\;Devices\;Price_{\$}}{Weighted\;Lifespan} \end{array}$$

(6) $$\begin{array}{l} FEES_{BTC}\;=\;\frac{Total\;Transaction\;Fees\;\left(BTC\right)}{Daily\;Transaction\;Fees} \end{array}$$

The new equations become:

(7) $$\begin{array}{l} COST_{\frac{\$}{day}}=\;H_{\frac{hash}{s}}\;*\;Eff_{\frac{J}{hash}}\;*\;CE_{\frac{\$}{kWh}}\;*\;24_{\frac{h}{day}}+MAN_{\$} \end{array}$$

(8) $$\begin{array}{l} PROFIT_{\frac{BTC}{day}}=BR_{BTC}\;*\;\left[\frac{3,600_{\frac{s}{h}}\;*\;24_{\frac{h}{day}}}{BTs}\right]+FEES_{BTC} \end{array}$$

Moreover, due to the equality 1 joule = 1 watt^*^second, Equation (7) could be expressed as follows:

(9) $$\begin{array}{l} COST_{\$/day}=\;H_{hash/s}\;*\;Eff_{\frac{W\;*\;s}{hash}}\;*\;CE_{\frac{\$}{kWh}}\;*\;24_{h/day}+MAN_{\$} \end{array}$$

By converting watt in kilowatt/hour, it can be written as:

(10) $$\begin{array}{l} COST_{\$/day}=\;H_{hash/s}\;*\;\frac{Eff_{\frac{W\;*\;s}{hash}}}{1000}\;*\;CE_{\frac{\$}{kWh}}\;*\;24_{h/day}+MAN_{\$} \end{array}$$

(11) $$\begin{array}{l} COST_{\$/day}=\;H_{hash/s}\;*\;Eff_{\frac{kWh\;*\;s}{hash}}\;*\;CE_{\frac{\$}{kWh}}\;*\;24_{h/day}+MAN_{\$} \end{array}$$

According to the competitive market economic theories, the ratio between the cost and profit functions must lead to the price under equilibrium condition (Equation 12):

(12) $$\begin{array}{l} P_{\$/BTC}=\;\frac{COST_{\frac{\$}{day}}}{PROFIT_{\frac{BTC}{day}}} \end{array}$$

A historical price below the one predicted by the model would force a miner out of the market, since he is operating in loss, but at the same time, the removal of its devices from the network increases others' marginal profits (competition decreases), and at the end, the system would return to equilibrium. On the other hand, a historical price higher than what predicted by the model attracts more miners, thus increasing the number of devices operating in the network and decreasing others' marginal profits (competition increases). Again, the system would return in balance (Hayes, 2015).

We must remark that the assumption of an energy price per hemisphere is not very realistic. In fact, for large consumers, energy price is contractually set differently for peak times and less busy times. There is a lot of variation in the energy price of mines in different countries and circumstances (see, for example, Iceland with its geothermal cheap energy as a cheap energy example; Soltani et al., 2019). Taking more variation around energy prices into account would probably add a wider range of BTC prices (de Vries, 2016); due to the difficulties on collecting comparable data, we adopted a simplified proxy of the cost of energy.

Table 1 presents the sources used to collect and compute the required information.

**Table 1:** Sources.

**Variables**		**Sources**
*P*_*hist$*_	Historical price in US dollar	https://Bitcoinvisuals.com
*H*_*hash*/*s*_	Hashrate	
*BR*_*BTC*_	Block reward	
*D*	Difficulty	
BT_*s*_	Block time	Computed using *D* and *H*_*hash*/*s*_
*FEES*_*BTC*_	Transaction fees	https://charts.Bitcoin.com/bch/
*CE*_*$*/*kWh*_	Cost of energy	Computed using data from: en.Bitcoin.it/wiki/Mining_hardware_comparison https://archive.org/web/
*MAN*_*$*_	Hardware maintaining cost	
*EFF*_*J*/*hash*_	Hardware energy efficiency	

*Source: Authors' elaboration*.

We start the analysis by constructing a hardware sample that evolves during a chosen time window (2010–2018), which is divided in semesters associated with the introduction of a specific device (Table 2).

**Table 2:** Hardware sample.

**TYPE**	**MODEL**	**TIME**	**EFF. (Mhash/J)**	**PRICE (USD)**	**LIFESPAN**	
					**Before** **′17**	**After** **′17**
GPU	ATI FirePro M5800	2 s. 2010	1.45	175	2,880	1,440
GPU	Sapphire Radeon 5750 Vapor-X	2 s. 2010	1.35	160	2,880	1,440
GPU	GTX460	2 s. 2010	1.73	200	2,880	1,440
GPU	FirePro V5800	1 s. 2011	2.08	469	2880	1,440
FPGA	Avnet Spartan-6 LX150T	2 s. 2011	6.25	995	1,010	505
FPGA	AMD Radeon 7900	1 s. 2012	10.40	680	1,010	505
FPGA	Bitcoin Dominator X5000	2 s. 2012	14.70	750	1,010	505
FPGA	X6500	1 s. 2013	23.25	989	1,010	505
ASIC	Avalon 1	2 s. 2013	107.00	1,299	540	270
ASIC	Bitmain AntMiner S1	1 s. 2014	500.00	1,685	540	270
ASIC	Bitmain AntMiner S2	2 s. 2014	900.00	2,259	540	270
ASIC	Bitmain AntMiner S3	1 s. 2015	1,300.00	1,350	540	270
ASIC	Bitmain AntMiner S4	2 s. 2015	1,429.00	1,400	540	270
ASIC	Bitmain AntMiner S5	1 s. 2016	1,957.00	1,350	540	270
ASIC	Bitmain AntMiner S5+	2 s. 2016	2,257.00	2,307	540	270
ASIC	Bitmain AntMiner S7	1 s. 2017	4,000.00	1,832	540	270
ASIC	Bitmain AntMiner S9	2 s. 2017	10,182.00	2,400	540	270
ASIC	Ebit E9++	1 s. 2018	10,500.00	3,880	540	270
ASIC	Ebit E10	2 s. 2018	11,100.00	5,230	540	270

*Source: Authors' elaboration*.

Since the first Bitcoin was traded, there has been an evolution of the devices used by miners. The first ones adopted were GPU (Graphical Processing Unit) and later FPGA (Field-Programmable Gate Array), but these days, only ASIC (Application-Specific Integrated Circuit) is suitable for mining purposes.

For each device model, we collect the efficiency, expressed in Mhash/J, and the dollar price at the release day.

Technical data were collected from the Wikipedia pages https://en.Bitcoin.it/wiki/Mining__hardware__comparison and https://en.Bitcoin.it/wiki/Non-specialized__hardware__comparison by using in addition the online archive https://archive.org/web/, which allows the recovery of different webpages at the date in which they were modified, enabling the comparison before and after reviews^5^.

Since only ASIC devices were created with specifications to mining purpose, there is homogeneity among FPGA and especially among GPU hardware. Due to this fact and considering the difficulty to recover the release prices, we make some simplified assumptions about them based on the information available online. This means that given the same computational power, we assume price homogeneity among devices when they were not available for specific models^6^.

Given the hardware sample, we construct a weights distribution matrix (Table A.3 in Supplementary Material) that represents the evolution of the devices used during each semester of the time window selected, which are replaced following a substitution rate that increases over time. In fact, until 2012, before FPGA took roots, it is equal to 0.05; until 2016, we set it equal to 0.1, and in the last 2 years of the analysis, it is equal to 0.15^7^.

All computations are based on this matrix; indeed, we multiplied it by a specific column of the hardware sample table to obtain the biannual Efficiency (Table A.4 in Supplementary Material) (J/Hash), Weighted Devices' Prices ($) (Table A.5 in Supplementary Material), and Weighted Lifespans (Table A.6 in Supplementary Material). Regarding this latter matrix, we made further assumptions on the device lifespans by implementing Abbatemarco et al. (2018) assumptions. Hence, we set a lifespan equal to 2,880 days for GPU, 1,010 days for FPGA, and 540 days for ASIC, but after 2017, due to a supposed market growth phase, we halved these numbers (Table 2).

To evaluate the cost of energy, we follow the assumptions suggested by the cited researchers and we divide the world into two parts relative to Europe: East and West, each one with a fix electricity price equal to 0.04 and 0.175 $/kWh, respectively. The weights' evolution of the mining pool is set up in 2010 equal to 0.7 for the West part and 0.3 for the East part and it changes progressively until reaching in 2018 a 0.2 for the West and 0.8 for the East. We obtained a biannual cost of energy evolution measured by $/kWh by multiplying the biannual weights to the electricity costs and summing up the value for the West and the East (Table A.7 in Supplementary Material).

At this point, to smooth the values across the time window, we take the differences between *biannualMAN*_*$*_, *biannualEFF*_*J*/*Hash*_, and *biannualCE*_*$*/*kWh*_ at time *t* and *t* – 1 and we divide these values by the number of days in each semester, obtaining *DeltaMAN, DeltaEFF*, and *DeltaCE* (Table A.8 in Supplementary Material). Starting the first day of the analysis with the first value of the biannual matrixes, we compute the final variables as follows:

(13) $$\begin{array}{l} MAN_{\$}\left(t\right)=\;MAN_{\$}\;\left(t-1\right)+DeltaMAN \end{array}$$

(14) $$\begin{array}{l} EFF_{\frac{J}{hash}}\left(t\right)=\;EFF_{J/hash}\;\left(t-1\right)+DeltaEFF \end{array}$$

(15) $$\begin{array}{l} CE_{\frac{\$}{kWh}}\left(t\right)=\;CE_{\frac{\$}{kWh}}\left(t-1\right)+DeltaCE \end{array}$$

## Main Outcomes

**Figure 1:**
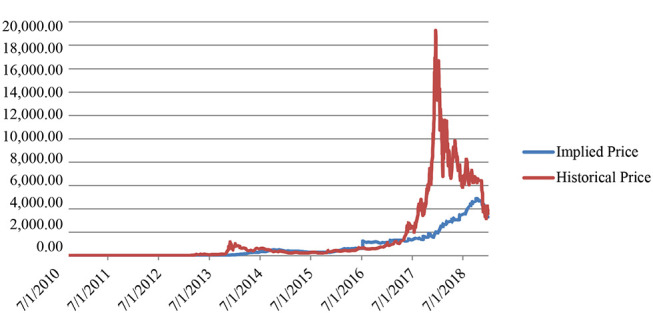
Historical market price vs. implied model price (July 2010–December 2018). Source: Authors' elaboration.

By applying Equations (8), (11), and (12), we obtain the model price^8^ and compare its evolution to the historical one (Figure 1).

The evolution of the model (or implied) price shows a spike during the second semester of 2016, probably because on 10th July 2016, the Block Reward halved from 25 to 12.5, leading to a reduction on the profit side and a consequent price increase.

Despite this episode, the historical price seems to fluctuate around the implied one until the beginning of 2017, the period in which Bitcoin price started raising exponentially, reaching its peak with a value equal to $19,270 on 19th December 2017. It declined during 2018, converging again to the model price.

Another divergence was detected at the end of 2013, but it was of a lower amount and resolved quickly.

Given the historical and implied price series, we make a further step than what Hayes (2019) and Abbatemarco et al. (2018) did, by including in the analysis a time frame even in the divergence phase. Therefore, we consider the period from 9th April 2014 to 31st December 2018. We select this time window also to base the analysis on solid data. Because of the difficulty to obtain reliable information on the hardware used in the mining process, we make some simplified assumptions on their features. By choosing this time window, we include the hardware sample whose data are more precise.

### Unit Root Tests

We first try to determine with different unit root tests whether the time series is stationary or not. The presence of a unit root indicates that a process is characterized by time-dependent variance and violates the weak stationarity condition^9^. We test the presence of a unit root with three procedures: the augmented Dickey–Fuller test (Dickey and Fuller, 1979), the Phillips–Perron test (Phillips and Perron, 1988), and the Zivot–Andrews test (Zivot and Andrews, 1992).

Given a time series {*y*_*t*_}, both the augmented Dickey–Fuller test (Dickey and Fuller, 1979) and the Phillips–Perron test are based on the general regression (Equation 16):

(16) $$\begin{array}{l} \Delta y_{t}=\alpha +\beta t+\theta y_{t-1}+\delta_{1}\Delta y_{t-1}+\ldots +\delta_{p-1}\Delta y_{t-p+1}+\varepsilon_{t} \end{array}$$

Where Δ*y*_*t*_ indicates changes in time series, α is the constant, *t* is the time trend, *p* is the order of the autoregressive process, and ε is the error term (Boffelli and Urga, 2016).

For both tests, the null hypothesis is that the time series contains a unit root; thus, it is not stationary (*H*_0_:θ = 0), while the alternative hypothesis asserts stationarity (*H*_0_:θ < 0).

Considering only the augmented Dickey–Fuller test, its basic idea is that if a series {*y*_*t*_} is stationary, then {Δ*y*_*t*_} can be explained only by the information included in its lagged values (Δ*y*_*t*−1_… Δ*y*_*t*−*p*+1_) and not from those in *y*_*t*−1_.

For each variable, we conduct this test firstly with a constant term and later by including also a trend^10^.

Table 3 presents the main findings of the test.

**Table 3:** Augmented Dickey–Fuller test.

	**Augmented Dickey–Fuller test**				
	**Constant**		**Constant** **+** **trend**		**Result**
	***t* stat**	***p*-value**	***t*-stat**	***p*-value**	
lnPrice	−0.606	0.8696	−1.839	0.6856	NO stationary
lnModelPrice	−0.467	0.8982	−1.669	0.7644	NO stationary
ΔlnPrice	−7.694	0.0000	−7.697	0.0000	Stationary
ΔlnModelPrice	−8.041	0.0000	−8.038	0.0000	Stationary
**Critical values**					
**Constant**			**Constant** **+** **trend**		
1%	5%	10%	1%	5%	10%
−3.430	−2.860	−2.570	−3.960	−3.410	−3.120

*Source: Authors' elaboration*.

The Phillips–Perron test points out that the process generating *y*_*t*_ might have a higher order of autocorrelation than the one admitted in the test equation. This test corrects the issue, and it is robust in case of unspecified autocorrelation or heteroscedasticity in the disturbance term of the equation. Table 4 displays the test results.

**Table 4:** Phillips–Perron test.

	**Phillips–Perron test**				
	**Constant**		**Constant** **+** **trend**		**Result**
	***t* stat**	***p*-value**	***t* stat**	***p*-value**	
lnPrice	−0.437	0.9037	−1.546	0.8130	NO stationary
lnModelPrice	−0.637	0.8624	−1.805	0.7021	NO stationary
ΔlnPrice	−34.394	0.0000	−34.385	0.0000	Stationary
ΔlnModelPrice	−42.972	0.0000	−42.959	0.0000	Stationary
**Critical values**					
**Constant**			**Constant** **+** **trend**		
1%	5%	10%	1%	5%	10%
−3.430	−2.860	−2.570	−3.960	−3.410	−3.120

*Source: Authors' elaboration*.

The main difference between these tests is that the latter applies Newey–West standard errors to consider serial correlation, while the augmented Dickey–Fuller test introduces additional lags of the first difference.

Since the previous tests do not allow for the possibility of a structural break in the series, Zivot and Andrews (1992) propose to examine the presence of a unit root including the chance of an unknown date of a break-point in the series. They elaborate three models to test for the presence of a unit root considering a one-time structural break:

a) permits a one-time change in the intercept of the series:

(17) $$\begin{array}{l} \Delta y_{t}=\alpha +\beta t+\theta y_{t-1}+\gamma DU_{t}+\delta_{1}\Delta y_{t-1}+\ldots \\ \;+\delta_{p-1}y_{t-p+1}+\varepsilon_{t} \end{array}$$

b) permits a one-time change in the slope of the trend function:

(18) $$\begin{array}{l} \Delta y_{t}=\alpha +\beta t+\theta y_{t-1}+\vartheta DT_{t}+\delta_{1}\Delta y_{t-1}+\ldots \\ \;+\delta_{p-1}\Delta y_{t-p+1}+\varepsilon_{t} \end{array}$$

c) combines the previous models:

(19) $$\begin{array}{l} \Delta y_{t}=\alpha +\beta t+\theta y_{t-1}+\gamma DU_{t}+\vartheta DT_{t}+\delta_{1}\Delta y_{t-1}+\ldots \\ \;+\delta_{p-1}\Delta y_{t-p+1}+\varepsilon_{t} \end{array}$$

Where *DU*_*t*_ is a dummy variable that relates to a mean shift at a given break-date, while *DT*_*t*_ is a trend shift variable.

The null hypothesis, which is the same for all three models, states that the series contains a unit root (*H*_0_:θ = 0), while the alternative hypothesis asserts that the series is a stationary process with a one-time break occurring at an unknown point in time (*H*_0_:θ < 0) (Waheed et al., 2006).

The results in Table 5 confirm what the other tests predict: both series are integrated of order 1. Since this last test identifies for Δ*lnPrice* the presence of a structural break on 18th December 2017 and after this date the Bitcoin price reaches its higher value to start declining later, we add to the analysis a dummy variable related to this observation, in order to take into account a broken linear trend in a series.

**Table 5:** Zivot–Andrews test.

	**Zivot–Andrews test**									
	**Intercept**			**Trend**			**Intercept** **+** **trend**			**Result**
	***t*** **stat**	**Break**	**Date**	***t*** **stat**	**Break**	**Date**	***t*** **stat**	**Break**	**Date**	
lnPrice	−2.964	1,083	26/03/2017	−2.049	261	25/12/2014	−2.562	1,196	17/07/2017	NON-stationary
lnModelPrice	−3.221	281	14/01/2015	−3.357	408	21/05/2015	−3.914	620	19/12/2015	NON-stationary
ΔlnPrice	−34.905	1,350	18/12/2017	−34.626	1,285	14/10/2017	−34.895	1,350	18/12/2017	Stationary
ΔlnModelPrice	−42.848	582	11/11/2015	−42.781	1,469	16/04/2018	−42.858	582	11/11/2015	Stationary

**Table:** 

**Critical values**								
**Intercept**			**Trend**			**Intercept** **+** **trend**		
1%	5%	10%	1%	5%	10%	1%	5%	10%
−5.34	−4.8	−4.58	−4.93	−4.42	−4.11	−5.57	−5.08	−4.82

*Source: Authors' elaboration*.

### Identifying the Number of Lags

The preferred lag length is the one that generates the lowest value of the information statistic considered. We follow Lütkepohl's intuition that “*the SBIC and HQIC provide consistent estimates of the true lag order, while the FPE and AIC overestimate the lag order with positive probability”* (Becketti, 2013). Therefore, for our analysis, we select 1 lag (Table 6)^11^.

**Table 6:** Proper number of lags.

**Lag**	**LL**	**LR**	***df***	***P***	**FPE**	**AIC**	**HQIC**	**SBIC**
0	7160.95				8.0e−07	−8.36581	−8.3611	−8.35308
1	7190.57	59.237	4	0.000	7.7e−07	−8.39575	−8.38633*	−8.37029*
2	7192.42	3.7134	4	0.446	7.8e−07	−8.39325	−8.37911	−8.35506
3	7194.48	4.1059	4	0.392	7.8e−07	−8.39097	−8.37231	−8.34005
4	7195.74	2.5346	4	0.638	7.8e−07	−8.38778	−8.36422	−8.32413
5	7197.81	4.1319	4	0.388	7.8e−07	−8.38552	−8.35725	−8.30914
6	7199.73	3.8486	4	0.427	7.8e−07	−8.38309	−8.35011	−8.29399
7	7201.63	3.8014	4	0.434	7.9e−07	−8.38064	−8.34295	−8.2788
8	7204.56	5.8468	4	0.211	7.9e−07	−8.37938	−8.33698	−8.26482
9	7208.36	7.6003	4	0.107	7.9e−07	−8.37914	−8.33204	−8.25185
10	7212.23	7.7429	4	0.101	7.9e−07	−8.37899	−8.32717	−8.23897
11	7213.48	2.5086	4	0.643	7.9e−07	−8.37578	−8.31925	−8.22304
12	7225.63	24.303	4	0.000	7.8e−07	−8.38531	−8.32407	−8.21983
13	7243.57	35.872*	4	0.000	7.7e−07*	−8.4016*	−8.33565	−8.2234
14	7244.29	1.4495	4	0.836	7.7e−07	−8.39777	−8.32711	−8.20684
15	7246.50	4.4025	4	0.354	7.7e−07	−8.39567	−8.3203	−8.19201
16	7248.86	4.7357	4	0.316	7.8e−07	−8.39376	−8.31368	−8.17737

*Source: Authors' elaboration*.

### Identifying the Number of Cointegrating Relationships

A cointegrating relationship is a relationship that describes the long-term link among the levels of a number of the non-stationary variables. Given *K* non-stationary variables, they can have at most *K* – 1 cointegrating relationships. Since we have only two non-stationary variables (*lnPrice* and *lnModelPrice*), we could obtain, at most, only one cointegrating relationship.

If series show cointegration, a VAR model is no more the best suited one for the analysis, but it is better to implement a Vector Error-Correction Model (VECM), which can be written as (20):

(20) $$\begin{array}{l} \Delta y_{t}=\mu +\delta t+\alpha \beta^{\prime }u_{t-1}+\overset{p-1}{\underset{i=1}{\sum }}\Gamma_{i}\Delta y_{t-i}+\varepsilon_{t} \end{array}$$

Where the deterministic components μ + δ*t* are, respectively, the linear and the quadratic trend in *y*_*t*_ that can be separated into the proper trends in *y*_*t*_ and those of the cointegrating relationship. This depends on the fact that in a first-difference equation: a constant term is a linear trend in the level of the variables (*y*_*t*_ = κ + λ*t* → Δ*y*_*t*_ = λ), while a linear trend derives from the quadratic one in the regression in levels ($y_{t}=\kappa +\lambda t+\omega t^{2}$ → Δ*y*_*t*_ = λ+2ω*t* − ω). Therefore, μ≡αν + γ, and δ*t* = α*ρt*+ τ*t*.

By substituting in the previous expression, the VECM can be expressed as Equation (21):

(21) $$\begin{array}{l} \Delta y_{t}=\alpha \left(\beta^{\prime }y_{t-1}+\nu +\rho t\right)+\overset{p-1}{\underset{i=1}{\sum }}\Gamma_{i}\Delta y_{t-i}+\gamma +\tau t+\varepsilon_{t} \end{array}$$

Where the first part $\alpha (\beta \prime u_{t-1}+\nu +\rho t)$ represents the cointegrating equations, while the second $\overset{p-1}{\underset{i=1}{\sum }}\Gamma_{i}\Delta y_{t-i}+\gamma +\tau t+\varepsilon_{t}$ refers to the variables in levels.

This representation allows specifying five cases that Stata tests:

1. Unrestricted trend: allows for quadratic trend in the level of *y*_*t*_(τ*t* appears in the equation) and states that the cointegrating equations are trend stationary, which means they are stationary around time trends.
2. Restricted trend (τ = 0): excludes quadratic trends but includes linear trends (ρ*t*). As in the previous case, it allows the cointegrating equations to be trend stationary.
3. Unrestricted constant (τ = 0, ρ = 0): lets linear trends in *y*_*t*_ to present a linear trend (γ) but the cointegrating equations are stationary around a constant means (ν).
4. Restricted constant (τ = 0, ρ = 0, γ = 0): rules out any trends in the levels of the data but the cointegrating relationships are stationary around a constant mean (ν).
5. No trend (τ = 0, ρ = 0, γ = 0, ν = 0): considers no non-zero means or trends.

Starting from these different specifications, the Johansen test can detect the presence of a cointegrating relationship in the analysis. The null hypothesis states, again, that there are no cointegrating relationships against the alternative that the null is not true. *H*_0_ is rejected if the trace statistic is higher than the 5% critical value.

We run the test with each case specification and the results agree to detect zero cointegrating equations (a maximum rank of zero). Only the unrestricted trend does not display any conclusion from the test but, since the other results matched, we consider *rank* = 0 the right solution. This implies that the two time series could be fitted into a VAR model.

### VAR Model

The VAR model allows investigating the interaction of several endogenous time series that mutually influence each other. We do not only want to detect if Bitcoin price could be determined by the one suggested by the cost of production model; we also want to check if the price has an influence on the model price. This latter relation can occur if, for example, a price increase leads to a higher cost for the mining hardware. In fact, a raise in the price represents also a higher reward if the mining process is successfully conducted, with the risk to push hardware price atop, which in turn could boost the model price up.

To explain how a VAR model is constructed, we present a simple univariate AR(*p*) model, disregarding any possible exogenous variables, which can be written as (22):

(22) $$\begin{array}{l} y_{t}=\mu +\phi_{1}y_{t-1}+\ldots +\phi_{p}y_{t-p}+\varepsilon_{t} \end{array}$$

Or, in a concise form (23):

(23) $$\phi (L)y_{t}=\mu +\varepsilon_{t}$$

where *y*_*t*_ depends on its *p* prior values, a constant (μ) and a random disturbance (ε_*t*_).

A vector of *n* jointly endogenous variables is express as (24):

(24) $$\begin{array}{l} y_{t}=\left[\begin{array}{c} y_{1,t}\\ y_{2,t}\\ \vdots \\ y_{n,t} \end{array}\right] \end{array}$$

This *n*-element vector can be rearranged as a function (Equation 25) of *n* constants, *p* prior values of *Y*_*t*_, and a vector of *n* random disturbances, ϵ_*t*_:

(25) $$\begin{array}{l} y_{t}=\mu +\phi_{1}y_{t-1}+\ldots +\phi_{p}y_{t-p}+\epsilon_{t} \end{array}$$

Where μ is a vector (Equation 26) of the *n*-constants:

(26) $$\begin{array}{l} \mu =\left[\begin{array}{c} \mu_{1}\\ \mu_{2}\\ \vdots \\ \mu_{p} \end{array}\right] \end{array}$$

the matrix of coefficients Φ_*i*_ is Equation (27):

(27) $$\begin{array}{l} \Phi_{1}=\left[\begin{array}{cccc} \phi_{i,11} & \phi_{i,12} & \cdots & \phi_{i,1n}\\ \phi_{i,21} & \phi_{i,22} & \cdots & \phi_{i,2n}\\ \vdots & \vdots & \ddots & \vdots \\ \phi_{i,n1} & \phi_{i,n2} & \ldots & \phi_{i,nn} \end{array}\right] \end{array}$$

and ϵ_*t*_ consists in Equation (28):

(28) $$\begin{array}{l} \epsilon_{t}=\left[\begin{array}{c} \varepsilon_{1}\\ \varepsilon_{2}\\ \vdots \\ \varepsilon_{p} \end{array}\right] \end{array}$$

With *Eϵ*_*t*_ = 0 and $E\epsilon_{t}{\epsilon^{\prime }}_{s}=\{\begin{array}{c} \Sigma ,\;t=s\\ 0,\;t\neq s \end{array}$

the elements of ϵ_*t*_ can be contemporaneously correlated.

Given these specifications, a *p*th-order VAR can be presented as Equation (29):

(29) $$\begin{array}{l} \Phi \left(L\right)u_{t}={\mu +\epsilon }_{t} \end{array}$$

To clarify this expression, the *i*th endogenous time series can be extracted from these basic VAR and be represented as (30):

(30) $$\begin{array}{l} y_{i,t}=\mu_{i}+\phi_{1,i1}y_{1,t-1}+\ldots +\phi_{1,in}y_{n,t-1}\\ \;+\phi_{2,i1}y_{1,t-2}+\ldots +\phi_{2,in}y_{n,t-2}+\ldots \\ \;+\phi_{p,i1}y_{1,t-p}+\ldots ++\phi_{p,in}y_{n,t-p}+\varepsilon_{i,t}\; \end{array}$$

The result of the VAR model considering the dummy variable is presented in Table 7:

**Table 7:** Regressions of the Vector Autoregression model.

**Variables**	**(1)**	**(2)**
	**dlnPrice**	**dlnModelPrice**
L.dlnPrice	0.18330223^***^	0.00799770
	(0.02359822)	(0.02055802)
L.dlnModelPrice	−0.00655017	−0.02899205
	(0.02762476)	(0.02406582)
Dummy	−0.00588960^***^	0.00027999
	(0.00185465)	(0.00161571)
Constant	0.00236755^***^	0.00149779^**^
	(0.00086910)	(0.00075713)
Observations	1,726	1,726
*R*^2^	0.04178812	0.00092579

*Standard errors in parentheses*.

*** *p < 0.01*,

** p < 0.05, and

* *p < 0.1*.

*Source: Authors' elaboration*.

As expected, the dummy is significant in the *dlnPrice* function but not in *dlnModelPrice*.

Looking at the significance of the parameters, we can see how *dlnPrice* depends on its lagged value, on the dummy and on the constant term, but it seems not to be linked with the lagged value of *dlnModelPrice*. The regression of *dlnModelPrice* appears not to be explained by any variable considered in the model. We then check the stationarity of the model. The results confirm that the model is stable and there is no residual autocorrelation (Table A.9 in Supplementary Material).

### Heteroscedasticity Correction

Given the series' path and the daily frequency of the data, the variables included in the model are probably heteroskedastic. This feature does not compromise the unbiasedness or the consistency of the OLS coefficients but invalidates the usual standard errors. In time series analysis, heteroscedasticity is usually neglected, as the autocorrelation of the error terms is seen as the main problem due to its ability to invalidate the analysis.

Since it is not possible to check and correct heteroscedasticity while performing the VAR model, we run each VAR regression separately and check the presence of heteroscedasticity by running the Breusch-Pagan test, whose null hypothesis states that the error variance are all equal (homoscedasticity) against the alternative hypothesis that the error variances change over time (heteroscedasticity).

(31) $$\begin{array}{l} H_{0}:\;\sigma_{1}^{2}=\sigma_{2}^{2}=\ldots =\sigma^{2}\; \end{array}$$

The null hypothesis is rejected if the probability value of the chi-square statistic (Prob < chi2) is < 0.05. The results of the test for both regressions show that the null hypothesis is always rejected, implying the presence of heteroscedasticity in the residuals (Table A.10 in Supplementary Material).

We try to correct the issue using heteroscedasticity-robust standard errors. The results are displayed in Table 8.

**Table 8:** Regressions with robust standard errors.

**Variables**	**(1)**	**(2)**
	**dlnPrice**	**dlnModelPrice**
L.dlnPrice	0.18330223^***^	0.00799770
	(0.04306718)	(0.01592745)
L.dlnModelPrice	−0.00655017	−0.02899205^***^
	(0.02681078)	(0.00979148)
Dummy	−0.00588960^***^	0.00027999
	(0.00225058)	(0.00142356)
Constant	0.00236755^***^	0.00149779^*^
	(0.00078480)	(0.00078942)
Observations	1,726	1,726
*R*^2^	0.04178812	0.00092579

*Robust standard errors in parentheses*.

*** *p < 0.01*,

** p < 0.05, and

* *p < 0.1*.

*Source: Authors' elaboration*.

These new robust standard errors are different from the standard errors estimated with the VAR model, while the coefficients are unchanged. The first difference of *lnPrice* depends even in this case on its lag, but, contrary from the VAR, now the first difference of *lnModelPrice* is not independent from its previous values. This new specification confirms the previous finding that each variable does not depend on the lagged value of the other one. Therefore, it seems that during the time window considered, the Bitcoin historical price is not connected with the price derived by Hayes' formulation, and vice versa.

Recalling Figure 1, it seems that the historical price fluctuated around the model (or implied) price until 2017, the year in which Bitcoin price significantly increased. During the last months of 2018, the prices seem to converge again, following a common path. In our analysis, we focus on the time window in which Bitcoin experienced its higher price volatility (Figure A.1 in Supplementary Material) and the results suggest that it is disconnected from the one predicted by the model. These findings may depend on the features of the new cryptocurrencies, which have not been completely understood yet.

The previous analyses, conducted on different time periods, by Hayes (2019) and Abbatemarco et al. (2018) assert that Bitcoin price could be justified by the costs and revenues of its blockchain network, leading to an opposite result from ours. We suggest that the difference could be based on the time window analyzed since we make a further step evaluating also the months in which Bitcoin price was pushed atop and did not follow a stable path. We think that there is not enough knowledge on cryptocurrencies to assert that Bitcoin price is (or is not) based on the profit and cost derived by the mining process, but these intrinsic characteristics must be considered and checked also in further analysis that include other possible Bitcoin price drivers suggested by the literature.

## Conclusions

The main findings of the analysis presented show how, in the considered time frame, the Bitcoin historical prices are not connected with the price derived from the model, and *vice versa*.

This result is different from the one obtained by Hayes (2019) and Abbatemarco et al. (2018), who conclude that the Bitcoin price could be explained by the cost of production model.

The reason behind these opposite outcomes could be the considered time window. In fact, our analysis includes also those months where Bitcoin price surges up, reaching a peak of $19,270 on 19th December 2017, without following a seasonal path (Figure A.1 in Supplementary Material). This has a relevant impact on the results even if the historical price started declining in 2018, converging again to the model one. Looking at the overall time frame, it seems that the increasing value of the historical price from the beginning of 2017 to the end of 2018 is a unique episode that required some months to get back to more standard behavior (Caporale et al., 2019).

It seems now possible to assert that Bitcoin could not be seen as a virtual commodity, or better not only. According to Abbatemarco et al. (2018), the implemented approach does not rule out the possibility of a bubble development and, given the actual time frame, this is the reason why it would be more precise to explain Bitcoin price not only with the one implied by the model, but also with other explanatory variables that the literature seems to identify as meaningful. Therefore, to avoid misleading results, Bitcoin intrinsic characteristics must be considered and checked by adding to the profit and cost functions also these suggested parameters that range from technical aspects and Internet components to financial indexes, commodity prices, and exchange rate. This could open new horizons for research, which, despite the traditional drivers, should consider also new factors such as Google Trends, Wikipedia queries, and Tweets. These elements are related to the Internet component and appear to be particularly relevant given the social and digital Bitcoin's nature.

Kristoufek's (2013) intuition, which considers Bitcoin as a unique asset that presents properties of both a speculative financial asset and a standard one, whose price drivers will change over time considering its dynamic nature and volatility, seems to be confirmed.

The explanatory power of the VAR specification we implemented to inspect fundamental vs. market price dynamics could be quite low, which is to ascribe to missing factors and volatility. Further researches could include more tests on the VAR specification also including other controls/factors to check whether, for example, the VIX is another and important explanatory factor. More involved analyses should also explore for latent factors and/or time-varying relationships with stochastic and jump components.

Although there are highlighted elements of uncertainty, Bitcoin has undoubtedly introduced to the market a new way to think about money transfers and exchanges. The distributed ledger technology could be a disruptive innovation for the financial sector, since it can ease communication without the need of a central authority. Moreover, the spread of private cryptocurrencies, which enter into competition with the public forms of money, could affect the monetary policy and the financial stability pursued by official institutions. For these reasons, central banks all over the world are seeking to understand if it is possible to adopt this technology in their daily operations, with the aim of including it in the financial system and controlling its implementations, enhancing its benefits, and reducing its risks (Gouveia et al., 2017; Bank for International Settlements, 2018).

## Data Availability Statement

All datasets generated for this study are included in the article/Supplementary Material.

## Author Contributions

FZ: Introduction, Literature Review, and Conclusions. CB: Materials and Methods, Main Outcomes, and Conclusions.

## Conflict of Interest

The authors declare that the research was conducted in the absence of any commercial or financial relationships that could be construed as a potential conflict of interest.

## Footnotes

^1^According to Abbatemarco et al. (2018), the nature of the variables considered suggests that they probably are mutually interdependent. Lütkepohl and Krätzig (2004) state that the analysis of interdependencies between time series is subject to the endogenous problem; part of the literature proposes to specify a Vector Auto Regressive model (VAR) that analyzes the causality between the two series estimated by the model. Engle and Granger (1987), instead, demonstrated that the estimate of such a model in the presence of non-stationary variables (i.e., with mean and variance non-constant over time) can lead to erroneous model specification and hence to unconditional regressions (spurious regressions). Scholars' intuition suggests that the price trend of a cryptocurrency and that of its estimated equilibrium prices are non-stationary time series, as there is a constant increase in their values over time.

^2^Results are shown in Table A.1 (Supplementary Material). In order to simplify the presentation, we display only the values for the last day of each month.

^3^Bitcoin could be obtained through both the mining process and the registration of transactions but, since Bitcoin supply is limited to 21 million, once it is reached, fees become the only remuneration source in the future.

^4^Fees computation results are displayed in Table A.1 (Supplementary Material).

^5^When possible, we double check Wikipedia prices with those on the websites of the companies producing mining hardware, and if they are not identical, we choose the latter.

^6^In detail, we approximate the prices of ATI FirePro M5800, Sapphire Radeon 5750 Vapor-X, GTX460, FireProV5800, Avnet Spartan-6 LX150T, and AMD Radeon 7900.

^7^Despite that ASIC devices have been released for the first time in 2013, they became the main devices used in the mining process only in 2015–2016. In the last 2 years of the analysis, we increase the substitution rate up to 0.15 because the competition among miners has been driven up as more sophisticated hardware was developed with a larger frequency.

^8^Table A.2 (Supplementary Material) displays all the variables required to compute the model price and compares it with the historical price. Since our time window involves 3,107 observation days, for the sake of simplicity, we present only the results for the last day of each month.

^9^The condition of weak stationarity asserts that *Var*(*r*_*t*_) = γ_*o*_, which means that the variance of the process is time invariant and equal to a finite constant.

^10^In order to select the proper number of lags to include in this test, we used, only for this part of the analysis, the open-source software Gretl. Its advantage is to apply clearly the Schwert criterion for the maximum lag (*p*_max_) estimation, which is given by: $p_{\max}=integer\;part\;of\;\left[12\;*\;{\left(\frac{T}{100}\right)}^{1/4}\right]$, where *T* is the number of observations. The test is conducted firstly with the suggested value of *p*_max_, but if the absolute value of the *t* statistic for testing the significance of the last lagged value is below the threshold 1.6, *p*_max_ is reduced by 1 and the analysis is recomputed. The process stops at the first maximum lag that returns a value >1.6. When this value is found, the augmented Dickey–Fuller test is estimated.

^11^To identify the proper lag length to be included in the VAR model, we use the “*varsoc*” command in Stata that displays a table of test statistics, which reports for each lag length, the log of the likelihood functions (LL), a likelihood-ratio test statistic with the related degrees of freedom and *p* value (LR, *df* , and *p*), and also four information criteria: Akaike's final prediction error (FPE); Akaike's information criterion (AIC), Hannan and Quinn's information criterion (HQIC), and Schwarz's Bayesian information criterion (SBIC). Every information criteria provide a trade-off between the complexity (e.g., the number of parameters) and the goodness of fit (based on the likelihood function) of a model. Since the output is sensitive to the maximum lag considered, we try different options by changing the one included in the command computation. We tried with 4, 8, 12, 16, 20, and 24 lags. After selecting a maximum lag length equal to 16, the optimal number of lags suggested changes: while the previous results agree recommending 1 lag with each information criteria, now the FPE and AIC diverge and propose 13 lags.
