# Supplementary material for: The Bitcoin as a Virtual Commodity: Empirical Evidence and Implications
Source: Front Artif Intell. 2020 Apr 30;3:21. doi: 10.3389/frai.2020.00021 (PMC7861307; doi:10.3389/frai.2020.00021)
Supplement: Supplementary file 1 [file Data_Sheet_1.zip › Table A1.pdf]

Table A.1: Block Time and Fees

DateTime	Hashrate	Difficulty	BT (s)	TOT Transaction Fees	Daily Transaction	Fees (BTC)
31/07/2010	2.464.398.497,64	244,213	425,6158	0,3	323	0,0009288
31/08/2010	5.701.927.397,06	623,387	469,5652	0,35	324	0,0010802
30/09/2010	9.570.510.422,87	1318,67	591,7808	0,0	976	0,0000000
31/10/2010	27.510.703.301,01	3091,737	482,6816	0,0	426	0,0000000
30/11/2010	68.953.790.963,51	6866,899	427,7228	0,03	456	6,58E-05
31/12/2010	116.641.878.937,44	14484,162	533,3333	0,0806039	565	0,0001427
31/01/2011	186.021.037.099,02	22012,381	508,2353	2,8210879	2.768	0,0010192
28/02/2011	414.505.489.254,17	55589,518	576	1,09	2.302	0,0004735
31/03/2011	689.209.762.451,73	68977,785	429,8507	4,0509	1.693	0,0023927
30/04/2011	1.030.377.325.650,24	109670,13	457,1429	7,7478269	3.632	0,0021332
31/05/2011	3.242.678.281.859,52	434877,05	576	18,242688	5.303	0,0034401
30/06/2011	13.094.963.916.948,80	1379192,3	452,356	20,008027	9.937	0,0020135
31/07/2011	14.709.605.099.479,30	1690895,8	493,7143	5,4575083	6.100	0,0008947
31/08/2011	13.697.915.570.338,70	1777774,5	557,4194	9,8799633	7.683	0,001286
30/09/2011	10.245.226.789.593,50	1689334,4	708,1967	3,3034121	5.388	0,0006131
31/10/2011	7.836.998.604.789,39	1203461,9	659,542	3,0873172	4.738	0,0006516
30/11/2011	8.783.602.827.814,50	1090715,7	533,3333	5,0343659	6.550	0,0007686
31/12/2011	8.706.721.615.131,83	1159929,5	572,1854	3,2551359	5.034	0,0006466
31/01/2012	9.751.129.414.143,54	1307728,4	576	28,379139	6.053	0,0046884
29/02/2012	12.246.527.873.312,90	1376302,3	482,6816	3,4994847	6.633	0,0005276
31/03/2012	13.745.590.058.278,80	1626553,5	508,2353	4,2526487	6.786	0,0006267
30/04/2012	13.423.639.484.651,40	1508589,7	482,6816	3,9405077	7.840	0,0005026
31/05/2012	10.914.801.619.952,20	1591075	626,087	19,028216	25.517	0,0007457
30/06/2012	11.329.307.771.943,70	1726566,6	654,5455	11,976852	23.222	0,0005158
31/07/2012	13.161.650.967.537,90	2036671,1	664,6154	20,1915	34.444	0,0005862
31/08/2012	16.985.500.288.894,50	2440642,6	617,1429	29,398627	34.045	0,0008635
30/09/2012	22.637.974.308.662,00	2864140,5	543,3962	13,885991	22.483	0,0006176
31/10/2012	23.653.504.403.772,50	3304356,4	600	23,812173	31.096	0,0007658
30/11/2012	21.368.636.454.168,60	3438909	691,2	28,942177	36.545	0,000792
31/12/2012	23.402.729.261.443,20	2979636,6	546,8354	33,267007	43.029	0,0007731
31/01/2013	28.482.674.089.138,00	2968775,3	447,6684	34,459397	41.090	0,0008386
28/02/2013	30.490.785.796.451,20	3651011,6	514,2857	57,564022	61.163	0,0009412
31/03/2013	56.251.874.756.664,30	6695826,3	511,2426	65,680689	60.832	0,0010797
30/04/2013	74.633.390.176.076,10	10076293	579,8658	45,746519	52.096	0,0008781
31/05/2013	105.726.140.279.199,00	12153412	493,7143	47,145952	56.516	0,0008342
30/06/2013	162.269.499.322.755,00	21335329	564,7059	24,446447	36.217	0,000675
31/07/2013	295.220.469.662.408,00	31256961	454,7368	38,709962	43.808	0,0008836
31/08/2013	728.865.067.652.167,00	65750060	387,4439	43,046084	61.944	0,0006949
30/09/2013	1.205.848.335.384.680,00	148819200	530,0613	25,451135	40.430	0,0006295
31/10/2013	4.100.400.017.728.000,00	390928788	409,4787	30,082821	49.095	0,0006127
30/11/2013	6.189.120.342.010.940,00	707408283	490,9091	25,947939	89.811	0,0002889
31/12/2013	10.801.537.922.461.200,00	1,181E+09	469,5652	11,692365	55.125	0,0002121
31/01/2014	20.066.445.524.103.800,00	2,194E+09	469,5652	13,333394	61.116	0,0002182

28/02/2014	28.158.518.665.937.100,00	3,13E+09	477,3481	14,685962	72.528	0,0002025
31/03/2014	44.800.630.179.722.400,00	5,007E+09	480	10,599807	54.971	0,0001928
30/04/2014	54.488.395.027.708.900,00	8,001E+09	630,6569	12,514559	64.708	0,0001934
31/05/2014	76.404.230.086.637.500,00	1,046E+10	587,7551	11,798537	65.979	0,0001788
30/06/2014	110.358.632.936.101.000,00	1,682E+10	654,5455	8,9302009	50.331	0,0001774
31/07/2014	143.434.608.511.561.000,00	1,874E+10	561,039	10,744746	65.667	0,0001636
31/08/2014	222.841.095.649.388.000,00	2,384E+10	459,5745	9,1553722	61.386	0,0001491
30/09/2014	263.623.410.749.534.000,00	3,466E+10	564,7059	12,353139	79.295	0,0001558
31/10/2014	336.304.905.509.895.000,00	3,599E+10	459,5745	13,717535	91.785	0,0001495
30/11/2014	320.532.059.789.074.000,00	4,03E+10	540	11,206979	80.187	0,0001398
31/12/2014	307.081.470.445.705.000,00	4,064E+10	568,4211	12,529827	83.173	0,0001506
31/01/2015	340.579.824.489.384.000,00	4,127E+10	520,4819	10,997977	79.874	0,0001377
28/02/2015	329.538.396.656.713.000,00	4,668E+10	608,4507	14,651859	103.737	0,0001412
31/03/2015	348.351.298.391.091.000,00	4,672E+10	576	15,038765	104.273	0,0001442
30/04/2015	317.142.355.890.591.000,00	4,761E+10	644,7761	16,907277	117.681	0,0001437
31/05/2015	359.082.535.929.914.000,00	4,881E+10	583,7838	16,07206	122.358	0,0001314
30/06/2015	356.089.184.959.351.000,00	4,94E+10	595,8621	18,812457	124.919	0,0001506
31/07/2015	392.414.063.077.807.000,00	5,228E+10	572,1854	34,198081	124.672	0,0002743
31/08/2015	423.446.540.478.744.000,00	5,426E+10	550,3185	19,542684	96.926	0,0002016
30/09/2015	436.537.293.643.837.000,00	5,934E+10	583,7838	27,498236	141.393	0,0001945
31/10/2015	454.915.351.205.089.000,00	6,225E+10	587,7551	31,187957	163.874	0,0001903
30/11/2015	585.641.183.501.102.000,00	7,272E+10	533,3333	22,430798	130.631	0,0001717
31/12/2015	733.276.604.663.887.000,00	1,04E+11	608,4507	31,016518	177.568	0,0001747
31/01/2016	1.080.007.040.825.580.000,00	1,20E+11	477,3481	39,032167	195.293	0,0001999
29/02/2016	1.080.919.494.946.580.000,00	1,63E+11	649,6241	45,836465	239.889	0,0001911
31/03/2016	1.020.134.644.775.590.000,00	1,65E+11	696,7742	45,017414	198.641	0,0002266
30/04/2016	1.296.655.181.852.460.000,00	1,79E+11	591,7808	49,381213	258.674	0,0001909
31/05/2016	1.258.297.790.411.960.000,00	1,99E+11	680,315	56,152944	206.139	0,0002724
30/06/2016	1.561.795.948.283.090.000,00	2,09E+11	576	61,677799	225.270	0,0002738
31/07/2016	1.634.366.548.269.600.000,00	2,13E+11	561,039	66,493384	199.563	0,0003332
31/08/2016	1.470.493.987.208.500.000,00	2,21E+11	644,7761	66,729189	248.782	0,0002682
30/09/2016	1.702.788.621.491.550.000,00	2,41E+11	608,4507	67,666403	241.285	0,0002804
31/10/2016	1.853.292.419.260.720.000,00	2,54E+11	587,7551	61,661948	205.888	0,0002995
30/11/2016	1.989.192.739.728.200.000,00	2,82E+11	608,4507	101,056	289.509	0,0003491
31/12/2016	2.337.271.653.891.100.000,00	3,18E+11	583,7838	99,824641	285.649	0,0003495
31/01/2017	2.832.474.910.223.240.000,00	3,93E+11	595,8621	138,07004	252.964	0,0005458
28/02/2017	3.352.427.031.868.180.000,00	4,41E+11	564,7059	198,13844	283.344	0,0006993
31/03/2017	3.352.999.966.358.030.000,00	5,00E+11	640	206,53209	286.240	0,0007215
30/04/2017	3.918.071.610.127.890.000,00	5,22E+11	572,1854	254,1149	342.452	0,000742
31/05/2017	4.858.244.024.949.570.000,00	5,96E+11	526,8293	589,40149	348.282	0,0016923
30/06/2017	5.448.313.580.843.890.000,00	7,12E+11	561,039	345,33975	232.242	0,001487
31/07/2017	6.200.470.684.210.650.000,00	8,60E+11	595,8621	144,59399	194.083	0,000745
31/08/2017	7.196.655.779.676.010.000,00	8,88E+11	530,0613	391,62567	275.899	0,0014195
30/09/2017	7.569.349.388.439.090.000,00	1,10E+12	626,087	115,05341	256.088	0,0004493
31/10/2017	9.966.505.886.699.330.000,00	1,45E+12	626,087	258,96021	316.229	0,0008189
30/11/2017	11.182.287.318.862.000.000,00	1,35E+12	517,3653	290,66332	399.046	0,0007284

31/12/2017	15.177.350.249.534.300.000,00	1,87E+12	530,0613	764,87176	338.192	0,0022616
31/01/2018	15.139.761.911.894.200.000,00	2,60E+12	738,4615	162,67806	239.721	0,0006786
28/02/2018	23.172.168.746.403.100.000,00	3,01E+12	557,4194	49,929181	215.529	0,0002317
31/03/2018	22.031.861.231.379.800.000,00	3,46E+12	675	25,665438	184.109	0,0001394
30/04/2018	30.990.335.301.692.100.000,00	4,02E+12	557,4194	35,451689	183.970	0,0001927
31/05/2018	36.396.936.182.652.700.000,00	4,31E+12	508,2353	22,629538	205.065	0,0001104
30/06/2018	35.588.942.295.590.800.000,00	5,08E+12	612,766	26,962117	194.781	0,0001384
31/07/2018	43.474.975.975.433.800.000,00	5,95E+12	587,7551	24,510049	231.194	0,000106
31/08/2018	51.833.889.753.678.900.000,00	6,73E+12	557,4194	23,998609	233.840	0,0001026
30/09/2018	57.956.171.195.079.300.000,00	7,15E+12	530,0613	19,399876	222.999	8,70E-05
31/10/2018	58.201.028.613.416.300.000,00	7,18E+12	530,0613	19,021598	287.918	6,61E-05
30/11/2018	33.404.486.801.782.100.000,00	6,65E+12	855,4455	44,471941	245.476	0,0001812
31/12/2018	39.345.457.366.303.000.000,00	5,11E+12	557,4194	13,277209	259.993	5,11E-05

Source: Authors’ elaboration
