# Supplementary material for: The Bitcoin as a Virtual Commodity: Empirical Evidence and Implications
Source: Front Artif Intell. 2020 Apr 30;3:21. doi: 10.3389/frai.2020.00021 (PMC7861307; doi:10.3389/frai.2020.00021)
Supplement: Supplementary file 1 [file Data_Sheet_1.zip › Table A2.pdf]

Table A.2: Model Price and Historical Price

Date Time	Historical Hashrate (H/s)	EFF (J/hash)	CE ($/kWh)	MAN ($)	BR (BTC)	BTs	Fees (BTC)	COST	PROFIT	Model Price	Historical Price
31/07/2010	2.464.398.497,64	6,59E-07	0,13381216	0,06278	50	425,6158	0,00092879	5,28	10.150,00	0	0,06
31/08/2010	5.701.927.397,06	6,57E-07	0,13310139	0,06367	50	469,5652	0,00108025	12,02	9.200,00	0	0,06
30/09/2010	9.570.510.422,87	6,54E-07	0,13241355	0,06453	50	591,7808	0,00000000	19,95	7.300,00	0	0,06
31/10/2010	27.510.703.301,01	6,51E-07	0,13170279	0,06542	50	482,6816	0,00000000	56,66	8.950,00	0,01	0,19
30/11/2010	68.953.790.963,51	6,48E-07	0,13101495	0,06628	50	427,7228	6,58E-05	140,55	10.100,00	0,01	0,21
31/12/2010	116.641.878.937,44	6,45E-07	0,13030418	0,06717	50	533,3333	0,00014266	235,36	8.100,00	0,03	0,29
31/01/2011	186.021.037.099,02	6,27E-07	0,12958201	0,07112	50	508,2353	0,00101918	363,08	8.500,00	0,04	0,51
28/02/2011	414.505.489.254,17	6,11E-07	0,12892939	0,07477	50	576	0,0004735	783,96	7.500,00	0,1	0,88
31/03/2011	689.209.762.451,73	5,93E-07	0,12820684	0,07882	50	429,8507	0,00239274	1.257,87	10.050,00	0,13	0,79
30/04/2011	1.030.377.325.650,24	5,76E-07	0,1275076	0,08274	50	457,1429	0,00213321	1.815,14	9.450,00	0,19	3,29
31/05/2011	3.242.678.281.859,52	5,58E-07	0,12678505	0,08679	50	576	0,00344007	5.501,68	7.500,00	0,73	8,88
30/06/2011	13.094.963.916.948,80	5,40E-07	0,12608581	0,0907	50	452,356	0,00201349	21.402,26	9.550,00	2,24	16,51
31/07/2011	14.709.605.099.479,30	5,16E-07	0,12537466	0,09664	50	493,7143	0,00089467	22.856,91	8.750,00	2,61	13,52
31/08/2011	13.697.915.570.338,70	4,93E-07	0,12466389	0,10264	50	557,4194	0,00128595	20.185,48	7.750,00	2,6	8,35
30/09/2011	10.245.226.789.593,50	4,69E-07	0,12397605	0,10844	50	708,1967	0,00061311	14.309,76	6.100,00	2,35	5,01
31/10/2011	7.836.998.604.789,39	4,46E-07	0,12326529	0,11444	50	659,542	0,00065161	10.329,72	6.550,00	1,58	3,19
30/11/2011	8.783.602.827.814,50	4,22E-07	0,12257745	0,12025	50	533,3333	0,00076861	10.915,61	8.100,00	1,35	2,91
31/12/2011	8.706.721.615.131,83	3,99E-07	0,12186668	0,12625	50	572,1854	0,00064663	10.149,19	7.550,00	1,34	4,6
31/01/2012	9.751.129.414.143,54	3,75E-07	0,12114835	0,1362	50	576	0,00468844	10.626,66	7.500,00	1,42	5,53
29/02/2012	12.246.527.873.312,90	3,53E-07	0,12047613	0,14563	50	482,6816	0,00052759	12.485,93	8.950,00	1,4	4,82
31/03/2012	13.745.590.058.278,80	3,29E-07	0,11975756	0,1557	50	508,2353	0,00062668	12.993,16	8.500,00	1,53	4,88
30/04/2012	13.423.639.484.651,40	3,06E-07	0,11906216	0,16546	50	482,6816	0,00050262	11.734,25	8.950,00	1,31	4,94
31/05/2012	10.914.801.619.952,20	2,82E-07	0,11834358	0,17554	50	626,087	0,00074571	8.747,93	6.900,00	1,27	5,15
30/06/2012	11.329.307.771.943,70	2,59E-07	0,11764818	0,18529	50	654,5455	0,00051576	8.292,15	6.600,00	1,26	6,64
31/07/2012	13.161.650.967.537,90	2,36E-07	0,11693716	0,21011	50	664,6154	0,00058621	8.726,62	6.500,00	1,34	9,24
31/08/2012	16.985.500.288.894,50	2,13E-07	0,11622639	0,23541	50	617,1429	0,00086352	10.106,41	7.000,00	1,44	10,19
30/09/2012	22.637.974.308.662,00	1,91E-07	0,11553855	0,2599	50	543,3962	0,00061762	11.996,08	7.950,00	1,51	12,35
31/10/2012	23.653.504.403.772,50	1,68E-07	0,11482779	0,2852	50	600	0,00076576	10.961,53	7.200,00	1,52	10,99
30/11/2012	21.368.636.454.168,60	1,46E-07	0,11413995	0,30969	25	691,2	0,00079196	8.543,69	3.125,00	2,73	12,51
31/12/2012	23.402.729.261.443,20	1,23E-07	0,11342918	0,33499	25	546,8354	0,00077313	7.836,98	3.950,00	1,98	13,44
31/01/2013	28.482.674.089.138,00	1,10E-07	0,11270701	0,37345	25	447,6684	0,00083863	8.457,79	4.825,00	1,75	20,53
28/02/2013	30.490.785.796.451,20	9,81E-08	0,11205439	0,40857	25	514,2857	0,00094116	8.045,52	4.200,00	1,92	32,67
31/03/2013	56.251.874.756.664,30	8,52E-08	0,11133184	0,44747	25	511,2426	0,00107971	12.806,64	4.225,00	3,03	92,59
30/04/2013	74.633.390.176.076,10	7,27E-08	0,1106326	0,48511	25	579,8658	0,00087812	14.408,91	3.725,00	3,87	138,98
31/05/2013	105.726.140.279.199,00	5,98E-08	0,10991005	0,524	25	493,7143	0,00083421	16.677,88	4.375,00	3,81	127,95
30/06/2013	162.269.499.322.755,00	4,73E-08	0,10921081	0,56164	25	564,7059	0,000675	20.120,62	3.825,00	5,26	94,26
31/07/2013	295.220.469.662.408,00	4,12E-08	0,10849966	0,62774	25	454,7368	0,00088363	31.703,67	4.750,00	6,67	104,48
31/08/2013	728.865.067.652.167,00	3,54E-08	0,10778889	0,69474	25	387,4439	0,00069492	66.751,11	5.575,00	11,97	136,3
30/09/2013	1.205.848.335.384.680,00	2,98E-08	0,10710105	0,75959	25	530,0613	0,00062951	92.217,33	4.075,00	22,63	133,44
31/10/2013	4.100.400.017.728.000,00	2,39E-08	0,10639029	0,82659	25	409,4787	0,00061275	250.371,13	5.275,00	47,46	205,74
30/11/2013	6.189.120.342.010.940,00	1,83E-08	0,10570245	0,89144	25	490,9091	0,00028892	286.757,64	4.400,00	65,17	1.103,89
31/12/2013	10.801.537.922.461.200,00	1,24E-08	0,10499168	0,95844	25	469,5652	0,00021211	338.195,63	4.600,00	73,52	749,09
31/01/2014	20.066.445.524.103.800,00	1,09E-08	0,10426951	1,096	25	469,5652	0,00021817	549.224,69	4.600,00	119,4	827,63
28/02/2014	28.158.518.665.937.100,00	9,72E-09	0,10361689	1,22236	25	477,3481	0,00020249	680.925,85	4.525,00	150,48	552,85
31/03/2014	44.800.630.179.722.400,00	8,38E-09	0,10289434	1,36227	25	480	0,00019283	927.201,32	4.500,00	206,04	458,66
30/04/2014	54.488.395.027.708.900,00	7,08E-09	0,1021951	1,49766	25	630,6569	0,0001934	946.314,38	3.425,00	276,3	445,96
31/05/2014	76.404.230.086.637.500,00	5,74E-09	0,10147255	1,63757	25	587,7551	0,00017882	1.067.613,98	3.675,00	290,51	616,81
30/06/2014	110.358.632.936.101.000,00	4,44E-09	0,10077331	1,77296	25	654,5455	0,00017743	1.184.483,61	3.300,00	358,93	621,19
31/07/2014	143.434.608.511.561.000,00	4,02E-09	0,10006216	1,8382	25	561,039	0,00016363	1.385.002,64	3.850,00	359,74	573,09
31/08/2014	222.841.095.649.388.000,00	3,63E-09	0,09935139	1,90095	25	459,5745	0,00014914	1.931.323,58	4.700,00	410,92	489,75
30/09/2014	263.623.410.749.534.000,00	3,26E-09	0,09866355	1,96168	25	564,7059	0,00015579	2.035.727,82	3.825,00	532,22	381,08
31/10/2014	336.304.905.509.895.000,00	2,88E-09	0,09795279	2,02443	25	459,5745	0,00014945	2.273.037,82	4.700,00	483,63	342,38
30/11/2014	320.532.059.789.074.000,00	2,50E-09	0,09726495	2,08515	25	540	0,00013976	1.871.660,48	4.000,00	467,92	378,9
31/12/2014	307.081.470.445.705.000,00	2,12E-09	0,09655418	2,1479	25	568,4211	0,00015065	1.505.284,24	3.800,00	396,13	315,19
31/01/2015	340.579.824.489.384.000,00	1,98E-09	0,09583201	2,20141	25	520,4819	0,00013769	1.550.292,53	4.150,00	373,56	228,53
28/02/2015	329.538.396.656.713.000,00	1,86E-09	0,09517939	2,24947	25	608,4507	0,00014124	1.402.856,28	3.550,00	395,17	253,52
31/03/2015	348.351.298.391.091.000,00	1,74E-09	0,09445684	2,30267	25	576	0,00014423	1.370.683,33	3.750,00	365,52	246,73

30/04/2015	317.142.355.890.591.000,00	1,61E-09	0,0937576	2,35416	25	644,7761	0,00014367	1.150.317,17	3.350,00	343,38	232,83
31/05/2015	359.082.535.929.914.000,00	1,48E-09	0,09303505	2,40736	25	583,7838	0,00013135	1.189.855,46	3.700,00	321,58	233,76
30/06/2015	356.089.184.959.351.000,00	1,36E-09	0,09233581	2,45885	25	595,8621	0,0001506	1.073.396,69	3.625,00	296,11	263,29
31/07/2015	392.414.063.077.807.000,00	1,29E-09	0,09162466	2,46025	25	572,1854	0,0002743	1.116.751,62	3.775,00	295,83	286,98
31/08/2015	423.446.540.478.744.000,00	1,23E-09	0,09091389	2,45992	25	550,3185	0,00020163	1.136.555,49	3.925,00	289,57	229,97
30/09/2015	436.537.293.643.837.000,00	1,17E-09	0,09022605	2,4596	25	583,7838	0,00019448	1.104.250,41	3.700,00	298,45	238,73
31/10/2015	454.915.351.205.089.000,00	1,10E-09	0,08951529	2,45927	25	587,7551	0,00019032	1.079.093,30	3.675,00	293,63	323,74
30/11/2015	585.641.183.501.102.000,00	1,04E-09	0,08882745	2,45894	25	533,3333	0,00017171	1.301.143,68	4.050,00	321,27	376,91
31/12/2015	733.276.604.663.887.000,00	9,78E-10	0,08811668	2,45861	25	608,4507	0,00017467	1.516.817,82	3.550,00	427,27	427,23
31/01/2016	1.080.007.040.825.580.000,00	9,39E-10	0,08739835	2,53563	25	477,3481	0,00019987	2.128.181,98	4.525,00	470,32	380,6
29/02/2016	1.080.919.494.946.580.000,00	9,04E-10	0,08672613	2,6101	25	649,6241	0,00019107	2.033.940,78	3.325,00	611,71	437,64
31/03/2016	1.020.134.644.775.590.000,00	8,66E-10	0,08600756	2,6897	25	696,7742	0,00022663	1.823.962,81	3.100,00	588,38	419,06
30/04/2016	1.296.655.181.852.460.000,00	8,30E-10	0,08531216	2,76673	25	591,7808	0,0001909	2.202.387,48	3.650,00	603,39	454,91
31/05/2016	1.258.297.790.411.960.000,00	7,92E-10	0,08459358	2,84633	25	680,315	0,0002724	2.022.549,30	3.175,00	637,02	536,74
30/06/2016	1.561.795.948.283.090.000,00	7,55E-10	0,08389818	2,92336	25	576	0,0002738	2.374.565,31	3.750,00	633,22	656,19
31/07/2016	1.634.366.548.269.600.000,00	7,08E-10	0,08318716	3,50106	12,5	561,039	0,0003332	2.311.261,70	1.925,00	1.200,66	637,11
31/08/2016	1.470.493.987.208.500.000,00	6,61E-10	0,08247639	4,09537	12,5	644,7761	0,00026822	1.924.777,31	1.675,00	1.149,12	575,59
30/09/2016	1.702.788.621.491.550.000,00	6,16E-10	0,08178855	4,6705	12,5	608,4507	0,00028044	2.058.030,90	1.775,00	1.159,45	607,31
31/10/2016	1.853.292.419.260.720.000,00	5,69E-10	0,08107779	5,26481	12,5	587,7551	0,00029949	2.050.763,15	1.837,50	1.116,06	703,38
30/11/2016	1.989.192.739.728.200.000,00	5,23E-10	0,08038995	5,83995	12,5	608,4507	0,00034906	2.007.692,39	1.775,00	1.131,09	743,19
31/12/2016	2.337.271.653.891.100.000,00	4,76E-10	0,07967918	6,43425	12,5	583,7838	0,00034947	2.127.820,45	1.850,00	1.150,17	960,06
31/01/2017	2.832.474.910.223.240.000,00	4,39E-10	0,07895701	6,65505	12,5	595,8621	0,00054581	2.356.652,68	1.812,50	1.300,22	944,21
28/02/2017	3.352.427.031.868.180.000,00	4,06E-10	0,07830439	6,84322	12,5	564,7059	0,00069929	2.557.532,97	1.912,50	1.337,27	1.179,77
31/03/2017	3.352.999.966.358.030.000,00	3,69E-10	0,07758184	7,05156	12,5	640	0,00072154	2.305.431,71	1.687,50	1.366,18	1.045,03
30/04/2017	3.918.071.610.127.890.000,00	3,34E-10	0,0768826	7,25318	12,5	572,1854	0,00074205	2.413.124,23	1.887,50	1.278,48	1.331,06
31/05/2017	4.858.244.024.949.570.000,00	2,97E-10	0,07616005	7,46152	12,5	526,8293	0,00169231	2.638.419,76	2.050,00	1.287,03	2.253,65
30/06/2017	5.448.313.580.843.890.000,00	2,62E-10	0,07546081	7,66314	12,5	561,039	0,00148698	2.581.552,37	1.925,00	1.341,07	2.519,27
31/07/2017	6.200.470.684.210.650.000,00	2,43E-10	0,07474966	7,80012	12,5	595,8621	0,00074501	2.699.406,28	1.812,50	1.489,33	2.787,33
31/08/2017	7.196.655.779.676.010.000,00	2,24E-10	0,07403889	7,93473	12,5	530,0613	0,00141945	2.868.460,96	2.037,50	1.407,83	4.689,86
30/09/2017	7.569.349.388.439.090.000,00	2,07E-10	0,07335105	8,06499	12,5	626,087	0,00044927	2.752.160,90	1.725,00	1.595,46	4.286,64
31/10/2017	9.966.505.886.699.330.000,00	1,88E-10	0,07264029	8,19959	12,5	626,087	0,0008189	3.269.542,29	1.725,00	1.895,39	6.248,49
30/11/2017	11.182.287.318.862.000.000,00	1,70E-10	0,07195245	8,32985	12,5	517,3653	0,0007284	3.290.459,48	2.087,50	1.576,27	9.947,53
31/12/2017	15.177.350.249.534.300.000,00	1,52E-10	0,07124168	8,46445	12,5	530,0613	0,00226165	3.945.345,24	2.037,50	1.936,36	13.205,27
31/01/2018	15.139.761.911.894.200.000,00	1,44E-10	0,07051951	9,02997	12,5	738,4615	0,00067861	3.677.978,07	1.462,50	2.514,86	10.044,73
28/02/2018	23.172.168.746.403.100.000,00	1,36E-10	0,06986689	9,55373	12,5	557,4194	0,00023166	5.290.600,85	1.937,50	2.730,63	10.684,50
31/03/2018	22.031.861.231.379.800.000,00	1,28E-10	0,06914434	10,13361	12,5	675	0,0001394	4.679.625,97	1.600,00	2.924,77	7.076,98
30/04/2018	30.990.335.301.692.100.000,00	1,20E-10	0,0684451	10,69478	12,5	557,4194	0,0001927	6.113.500,47	1.937,50	3.155,35	9.275,36
31/05/2018	36.396.936.182.652.700.000,00	1,12E-10	0,06772255	11,27466	12,5	508,2353	0,00011035	6.621.116,56	2.125,00	3.115,82	7.554,11
30/06/2018	35.588.942.295.590.800.000,00	1,04E-10	0,06702331	11,83583	12,5	612,766	0,00013842	5.954.819,71	1.762,50	3.378,62	6.387,05
31/07/2018	43.474.975.975.433.800.000,00	1,04E-10	0,06683696	11,87825	12,5	587,7551	0,00010602	7.227.128,45	1.837,50	3.933,13	7.981,32
31/08/2018	51.833.889.753.678.900.000,00	1,04E-10	0,06666848	11,90276	12,5	557,4194	0,00010263	8.584.395,67	1.937,50	4.430,66	6.977,97
30/09/2018	57.956.171.195.079.300.000,00	1,03E-10	0,06650544	11,92648	12,5	530,0613	8,70E-05	9.563.447,82	2.037,50	4.693,72	6.599,64
31/10/2018	58.201.028.613.416.300.000,00	1,03E-10	0,06633696	11,95099	12,5	530,0613	6,61E-05	9.567.716,85	2.037,50	4.695,81	6.328,46
30/11/2018	33.404.486.801.782.100.000,00	1,03E-10	0,06617391	11,9747	12,5	855,4455	0,00018117	5.471.359,20	1.262,50	4.333,75	4.045,47
31/12/2018	39.345.457.366.303.000.000,00	1,03E-10	0,06600544	11,99921	12,5	557,4194	5,11E-05	6.420.086,82	1.937,50	3.313,59	3.806,18

Source: Authors' elaboration
