# Supplementary figures and images for: The Bitcoin as a Virtual Commodity: Empirical Evidence and Implications
Source: Front Artif Intell. 2020 Apr 30;3:21. doi: 10.3389/frai.2020.00021 (PMC7861307; doi:10.3389/frai.2020.00021)

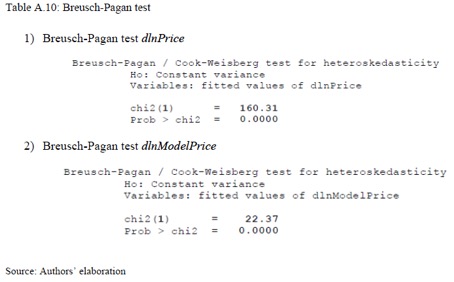

Supplement: Supplementary file 1 [file Data_Sheet_1.zip › Table A10.jpeg]

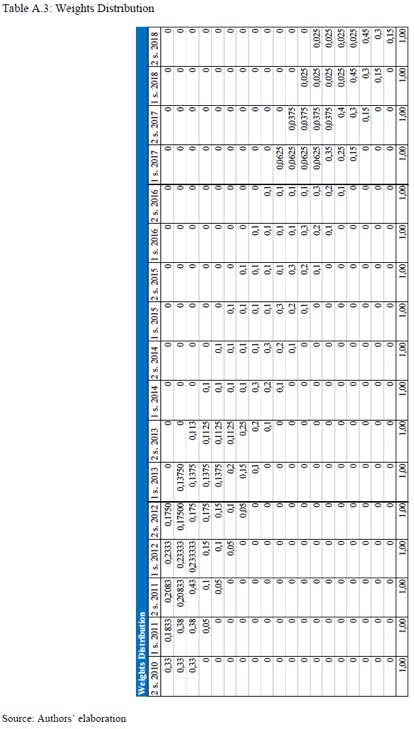

Supplement: Supplementary file 1 [file Data_Sheet_1.zip › Table A3.jpeg]

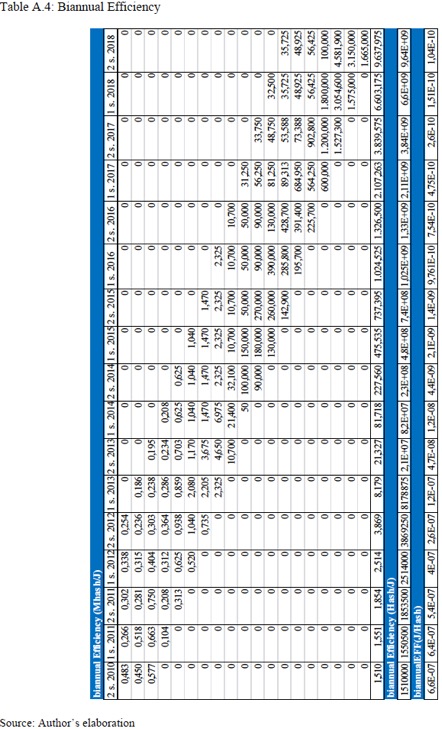

Supplement: Supplementary file 1 [file Data_Sheet_1.zip › Table A4.jpeg]

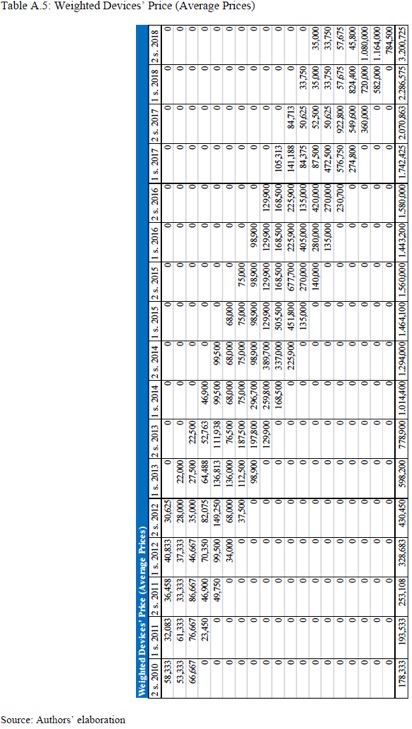

Supplement: Supplementary file 1 [file Data_Sheet_1.zip › Table A5.jpeg]

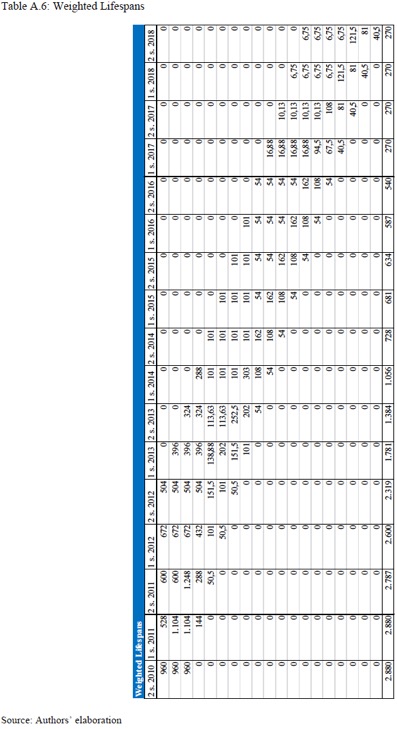

Supplement: Supplementary file 1 [file Data_Sheet_1.zip › Table A6.jpeg]

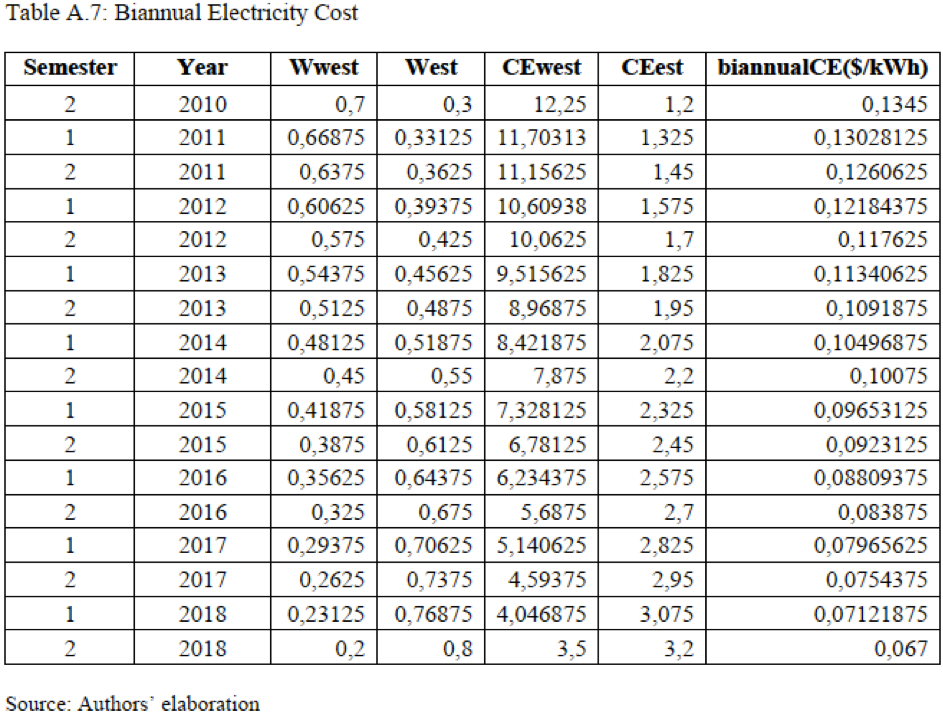

Supplement: Supplementary file 1 [file Data_Sheet_1.zip › Table A7.png]

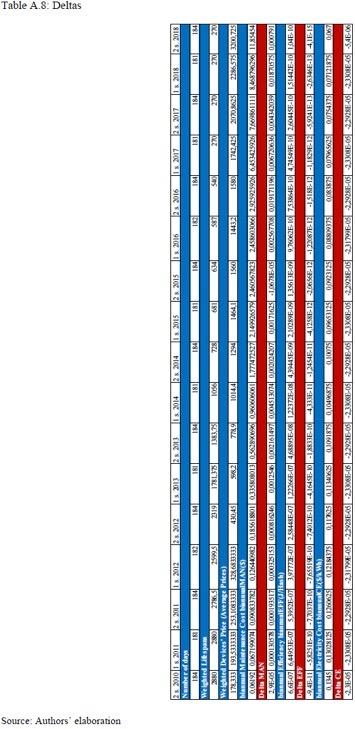

Supplement: Supplementary file 1 [file Data_Sheet_1.zip › Table A8.jpeg]

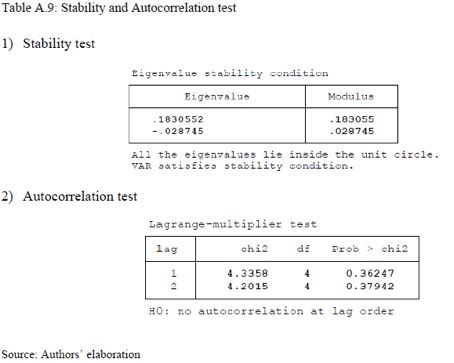

Supplement: Supplementary file 1 [file Data_Sheet_1.zip › Table A9.jpeg]

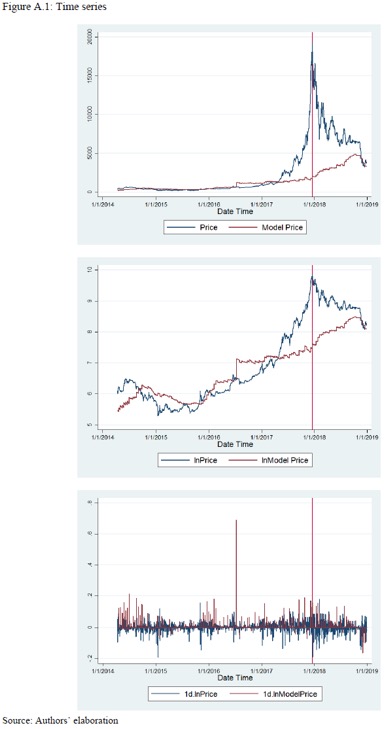

Supplement: Supplementary file 2 [file Image_1.jpeg]
